# AllerTOP - a server for *in silico *prediction of allergens

**DOI:** 10.1186/1471-2105-14-S6-S4

**Published:** 2013-04-17

**Authors:** Ivan Dimitrov, Darren R Flower, Irini Doytchinova

**Affiliations:** 1 Faculty of Pharmacy, Medical University of Sofia, 2 Dunav st., 1000 Sofia, Bulgaria; 2 Life and Health Sciences, Aston University, Aston Triangle, Birmingham, B4 7ET, UK

## Abstract

**Background:**

Allergy is a form of hypersensitivity to normally innocuous substances, such as dust, pollen, foods or drugs. Allergens are small antigens that commonly provoke an IgE antibody response. There are two types of bioinformatics-based allergen prediction. The first approach follows FAO/WHO *Codex alimentarius *guidelines and searches for sequence similarity. The second approach is based on identifying conserved allergenicity-related linear motifs. Both approaches assume that allergenicity is a linearly coded property. In the present study, we applied ACC pre-processing to sets of known allergens, developing alignment-independent models for allergen recognition based on the main chemical properties of amino acid sequences.

**Results:**

A set of 684 food, 1,156 inhalant and 555 toxin allergens was collected from several databases. A set of non-allergens from the same species were selected to mirror the allergen set. The amino acids in the protein sequences were described by three *z*-descriptors (*z_1_*, *z_2 _*and *z_3_*) and by auto- and cross-covariance (ACC) transformation were converted into uniform vectors. Each protein was presented as a vector of 45 variables. Five machine learning methods for classification were applied in the study to derive models for allergen prediction. The methods were: discriminant analysis by partial least squares (DA-PLS), logistic regression (LR), decision tree (DT), naïve Bayes (NB) and *k *nearest neighbours (*k*NN). The best performing model was derived by *k*NN at *k *= 3. It was optimized, cross-validated and implemented in a server named AllerTOP, freely accessible at http://www.pharmfac.net/allertop. AllerTOP also predicts the most probable route of exposure. In comparison to other servers for allergen prediction, AllerTOP outperforms them with 94% sensitivity.

**Conclusions:**

AllerTOP is the first alignment-free server for *in silico *prediction of allergens based on the main physicochemical properties of proteins. Significantly, as well allergenicity AllerTOP is able to predict the route of allergen exposure: food, inhalant or toxin.

## Background

Allergy is a form of hypersensitivity to normally innocuous substances, such as dust, pollen, foods or drugs. Allergies are more common in industrialized countries and in urban populations than in agricultural countries and in rural populations [1]. Allergens are small antigens that commonly provoke an IgE antibody response. Such antigens normally enter the body at very low doses by diffusion across mucosal surfaces, triggering a Th2 response [2]. The allergen-specific Th2 cells drive allergen-specific B cells to produce IgE, which binds to the high-affinity surface receptor FcεRI, present on mast cells, basophils, and activated eosinophils. On activation, these cells release stored mediators, which in turn give rise to inflammation and tissue damage causing a variety of symptoms. Inhalant allergens induce rhinitis, conjunctivitis and asthmatic symptoms, while food allergens lead to abdominal pain, bloating, vomiting and diarrhea. Food allergens rarely cause respiratory reactions and inhalant allergens seldom affect the gut [3].

Relatively few proteins act as allergens. Allergen proteins contain both T-cell epitopes capable of inducing Th2-type responses and B-cell epitopes to which IgE can bind. Allergens are also often proteins or glycoproteins with enzymatic activity, are resistant to proteolysis in the gut, are exceptionally heat stable, and are thought to be ovoid in shape [4]. Recently, it was found that allergen proteins have no or few bacterial homologues, in contrast to randomly selected control non-antigen proteins [5].

Although there is no consensus allergen structure, the Food and Agriculture Organization (FAO) and the World Health Organization (WHO) have produce *Codex alimentarius *guidelines for evaluating the potential allergenicity of novel proteins [6-8]. According to these guidelines, a query protein is potentially allergenic if it has either an identity of 6 to 8 contiguous amino acids or greater than 35% sequence similarity over a window of 80 amino acids when compared with known allergens.

Currently, there are two types of bioinformatics-based allergen prediction. The first approach follows FAO/WHO guidelines and searches for sequence similarity. The Structural Database of Allergenic Proteins [9], Allermatch [10] and AllerTool [11] all contain extensive databases of known allergen proteins and use them in sequence searches of query protein. Despite the high *sensitivity *(true positives/(true positives + false negatives)) of these methods, they are known to produce many false positives and have low *precision *or *positive predictive value *(true positives/(true positives + false positives)). Additionally, the discovery of novel antigens will be restricted by their lack of similarity to known allergens.

The second approach is based on identifying conserved allergenicity-related linear motifs. Stadler and Stadler defined 52 allergen motifs by comparing allergens to non-allergens using MEME [12]. Li *et al*. identified allergenic motifs by clustering known allergens, followed by wavelet analysis, and hidden Markov model (HMM) profile preparation of each identified motif [13]. Björklund *et al*. developed a detection method with used an Automated Selection of Allergen-Representative Peptides (DASARP) protocol [14]. AlgPred is a server for allergenic protein prediction which combines four methods for motif search: Support Vector Machines (SVM), MEME/MAST, IgE epitopes and Allergen-Representative Peptides (ARP) [15]. Both approaches assume that allergenicity is a linearly coded property.

Apart from T-cell epitopes able to induce Th2-type responses, allergen proteins must contain B-cell epitopes to which IgE can bind [4]. B-cell epitopes are discontinuous, conformational epitopes, arranged on the protein surface. Furmonaviciene *et al*. have defined allergen-specific patches consisting of an unusually high proportion of surface-exposed hydrophobic residues [16]. This finding is in a good agreement with the notion that the innate immune system has evolved to detect hydrophobic portions of immunogenic proteins comprising strings of aliphatic or aromatic amino acids [17].

Obviously, allergenicity, like antigenicity or immunogenicity, is a property encoded within a sequence in a subtle and possibly concealed manner: thus alignment-based approaches may not be able to detect properties, such as allergenicity, in an unambiguous manner. Here, we describe an alignment-independent method based on the auto- and cross-covariance (ACC) transformation of protein sequences into uniform, equal-length vectors. ACC is an protein sequence analysis method developed by Wold and colleagues [18], which has been applied to quantitative structure-activity relationships (QSAR) studies of peptides with different length [19,20], and for protein classification [21]. This method was used to identify immunoprotective proteins from various microbial organisms, including bacteria, viruses, parasites and fungi, as well as tumours [22]. The ACC transformation accounts for neighbour effects, i.e. the lack of independence between different sequence positions.

In the present study, we applied ACC pre-processing to sets of known allergens with different origins and routes of exposure, developing alignment-independent models for allergen recognition based on the main chemical properties of amino acid sequences. A mirror set of non-allergens was compiled from the same species. The principal properties of the 20 biogenic amino acids are represented by *z *descriptors, originally derived by Hellberg et al. [23]. They describe amino acid hydrophobicity, molecular size and polarity. Five machine learning methods (discriminant analysis by partial least squares DA-PLS, logistic regression LR, decision tree DT, naïve Bayes NB and *k *nearest neighbours *k*NN) were applied to discriminate between allergens and non-allergens. The best performing models were implemented in a server for allergen prediction, named AllerTOP. It is freely accessible via the World Wide Web at: http://www.pharmfac.net/allertop. The methodology described below is the first alignment-free bioinformatics tool for *in silico *identification of allergens. Additionally, it is able to predict their route of exposure: food, inhalant or toxin.

## Results

### Alignment-free presentation of the protein sequences

A set of 684 food, 1,156 inhalant and 555 toxin (venom or salivary) allergens was collected from several databases as described in Methods. A set of non-allergens from the same species were selected using a BLAST search tailored to mirror the allergen set. Non-allergens were selected as proteins with no sequence identity to known allergens, at an E-value of 0.001.

Twenty two of the food allergens were also toxins, 16 food allergens were also inhalant and 147 inhalant allergens were also toxins. The total set of proteins consisted of 2,210 allergens and 2,210 non-allergens.

The allergens and non-allergens were compared in terms of amino acid composition and main physico-chemical properties, including number of residues, molecular weight, extinction coefficient, iso-electric point, net charge at pH 7, and estimated water solubility. Data are given as Additional file 1. On this basis, no significant differences between allergens and non-allergens exist.

For the 5-fold cross-validation (5CV), each subset was divided randomly into 5 training (80%) and 5 test (20%) sets. Training sets were used to derive the models, while test sets were used to validate them. The total set was also divided into 5 training (3,536 proteins) and 5 test (884 proteins) sets.

The amino acids in the protein sequences were described by three *z*-descriptors (*z_1_*, *z_2 _*and *z_3_*). The descriptor *z_1 _*reflects the hydrophobicity of amino acids, the descriptor *z_2 _*reflects their size, and the descriptor *z_3 _*their polarity. The proteins were transformed into uniform vectors as described in Methods. Each protein was represented by a vector of 45 variables.

### Choice of a method for allergen prediction

Five supervised machine learning methods were applied separately to the subsets and to the total training set to derive models for allergen prediction. The models were validated by the corresponding test sets. The methods used in the study were discriminant analysis by partial least squares (DA-PLS), logistic regression (LR), decision tree (DT), naïve Bayes (NB) and *k *nearest neighbours (*k*NN) with *k *= 3.

**Figure 1:**
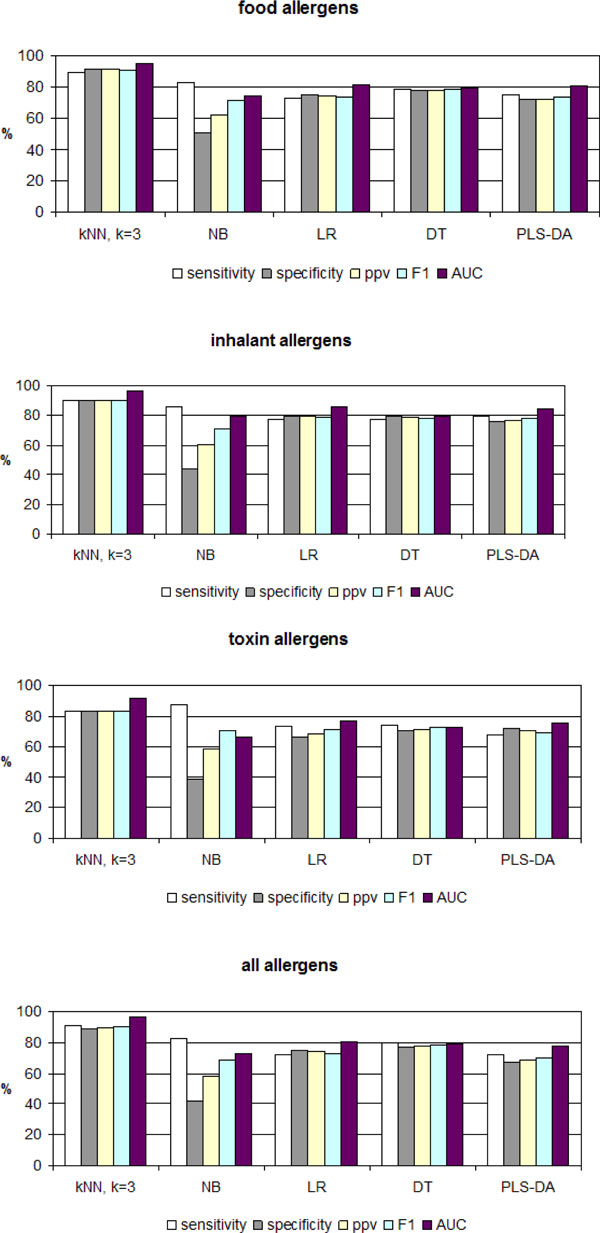
**Performance of the models derived by several machine learning algorithms on the origin test sets and on the total test set (*n *= 884)**. Abbreviations: *k*NN - nearest neighbours with *k *= 3, NB - naïve Bayes, LR - logistic regression, DT - decision tree, DA-PLS - discriminant analysis by partial least squares.

The performance of the derived models was assessed by 5CV using *sensitivity*, *specificity *and *positive predictive value (ppv) *and *F-measure (F1 *= 2 * sensitivity * *ppv*/(*sensitivity *+ *ppv*) at threshold 0.5, and *area under ROC curve (AUC) *(Figure 1). In all cases, the best performing model was *k*NN.

**Figure 2:**
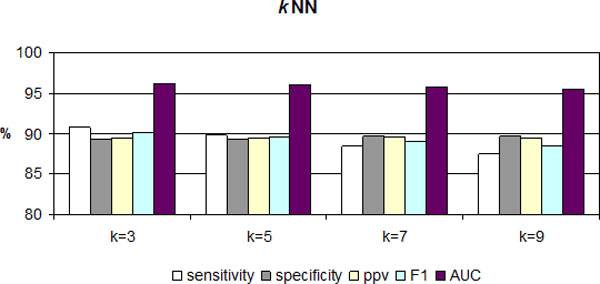
**Performance of *k*NN algorithm at different *k *values**. As an optimal number for *k *were selected 3.

Further, *k*NN models with different *k *values were derived and tested by 5CV to find the best *k *value. The results are shown in Figure 2. As *k *increases, the *specificity *of prediction slightly increases, while *sensitivity, F1 *and *AUC *decrease slightly, and *ppv *does not change. The optimal value for *k *was found to be 3.

### Cross comparative analysis of the models

**Figure 3:**
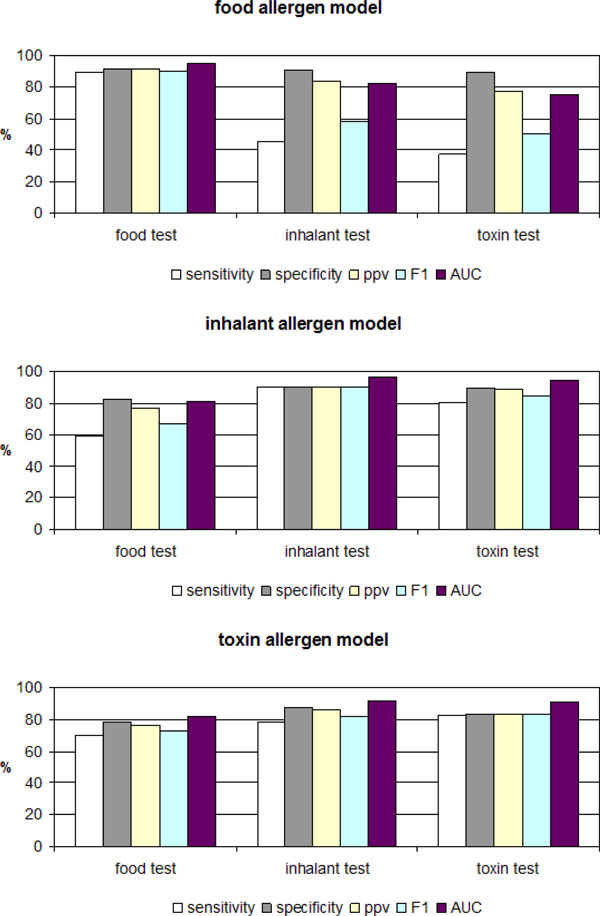
**Cross comparative analysis of the models for allergen prediction**.

Models were validated using the corresponding test sets (Figure 3). Additionally, cross comparative analysis was applied: the food allergen model was tested on inhalant and toxin test sets; the inhalant model was tested on food and toxin test sets; and the toxin model was tested on food and inhalant test sets.

The results showed that the route of exposure of the test set had no significant effect on *specificit*y, but *sensitivity *is clearly dependent on it. The low *sensitivities *of food and inhalant models cross tested on the corresponding test sets is in a good agreement with the observation that food allergens rarely cause respiratory reactions and inhalant allergens rarely affect the gut [3]. However, a good correlation exists between inhalant and toxin models. Such a correlation has not been observed before. One possible explanation could be the great number of common allergens between inhalant and toxin sets: 147 inhalant allergens are also toxins.

### AllerTOP server

The model based on the total set of allergens and non-allergens derived by the *k*NN algorithm with *k *= 3 and 5-fold cross-validated was made freely accessible via a server, named AllerTOP. AllerTOP is implemented in Python, with a GUI written in HTML. Protein sequences are uploaded in plain format. The results page returns the allergen status: "Probable Allergen" or "Probable Non-allergen". It also returns the *k *nearest neighbours in the training set. On this basis, AllerTOP defines the most probable route of exposure of tested proteins predicted as an allergen. The AllerTOP server also contains the datasets used in this study.

**Figure 4:**
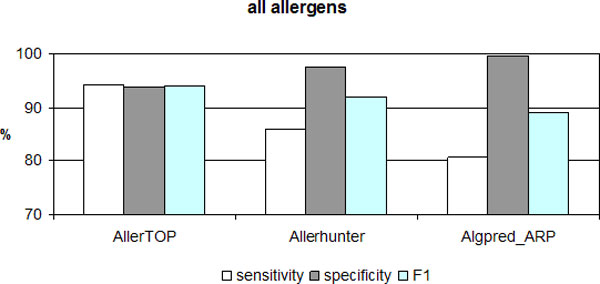
**The performance of web servers on the total set of 2,210 allergens and 2,210 non-allergens after 5-fold cross-validation**.

The performance of AllerTOP was compared to the freely available web servers using the total set of 2,210 allergens and 2,210 non-allergens (Figure 4). The servers accessible by the time of evaluations (December 2011) were AllerHunter [24] and AlgPred [15]. A short description of these servers is given in Methods. AllerHunter did not recognize 91 proteins because they are shorter than 20 amino acids. AlgPred did not recognize 15 proteins because they are shorter than 5 amino acids. AlgPred uses four different algorithms. The best performing of them is Algpred ARP and only it was considered in the comparison. Servers were compared in terms of *sensitivity*, *specificity *and *F1 *after 5CV. *Ppv *is not applicable because of differences in the number of tested proteins. *AUC *also is not used as there is no option to change threshold in AlgPred and AllerHunter.

The highest *sensitivity *was achieved by AllerTOP (94%), followed by Allerhunter (86%) and Algpred ARP (81%). Algpred ARP has the highest *specificity *(100%), closely followed by Allerhunter (98%) and AllerTOP (94%). Measuring *F1*, AllerTOP has the highest value (94%), then Allerhunter (92%) and Algpred ARP (89%). Additionally, AllerTOP gave the most balanced predictions.

## Discussion

Because of the high current incidence of allergenicity, with serious outcomes in many cases, the accurate prediction of allergenicity for new proteins originating from genetically modified crops or developed as protein drugs is crucial. Allergenicity is not straightforward to predict using alignment-based methods, since it is a phenomenon mediated by specific IgE antibodies requiring the presence of non-linear (conformational) B-cell epitopes in allergens. Here, we apply an alignment-independent method for protein presentation based on the main physicochemical properties of proteins that seeks to circumvent such issues. Our method uses *z*-descriptors to represent amino acids in the protein sequences, and an ACC transformation for conversion of protein sequences into uniform vectors.

In this paper, five well known and widely used methods of supervised machine learning were tested to discriminate between allergens and non-allergens. One of them (*k*NN) has been used before to predict food protein allergenicity [25]; the other four (DA-PLS, LR, DT and NB) are, to the best of our knowledge, applied for the first time to allergenicity prediction. DA-PLS has been used to predict immugenicity [22]. LR is a type of regression which delivers a class variable. DT is a tree-like graph of decisions which predicts a class outcome. The NB classifier assumes that the presence or absence of a particular structure descriptor is unrelated to the presence or absence of any other descriptor and derives an outcome based on maximum similarity. In the present study, the *k*NN clustering method had the best performance among the other algorithms for classification. The *k*NN algorithm was optimized and its performance was compared to other web servers for allergenicity prediction. The algorithm was implemented as a web server, freely accessible *via *http://www.pharmfac.net/allertop.

Allergens can enter the body via different routes: gut, respiratory system, skin, blood. The route of exposure determines the type and location of the atopic reaction: food allergens rarely cause respiratory reactions and inhalant allergens seldom affect the gut [3]. This observation is consistent with the results of our analysis. The food allergen-based model does not predict inhalant and toxin allergens. However, because of the many commonalities amongst allergens, some with multiple routes of exposure, the inhalant allergen-based model predicts toxins well and *vise versa*, the toxin model predicts inhalant allergens well. The *k*NN method allows us to predict the route of exposure with some certainty, based on the routes of exposure of the three nearest neighbours amongst known allergens.

The comparison of AllerTOP to other state-of-the-art servers for allergenicity prediction shows slight differences in their ability to distinguish between allergens and non-allergens. The high *sensitivity *of AllerTOP could be explained by its ability to identify new allergens, structurally diverse when compared to known allergens. The comparatively low *specificity *of AllerTOP may be due to the restricted number of non-allergens used to train the algorithm. Since non-allergenicity is often assumed rather than proven experimentally, other methods may be over-trained, possibly missing many putative allergens; and so our use of more conservative data-sets, and the lower concomitant specificity, is potentially a strength rather than a weakness. Moreover, the combined application of several methods for allergenicity prediction is able to achieve a successful prediction in the range of 94 - 100%.

## Conclusions

An alignment-free method for *in silico *prediction of allergens based on the main physicochemical properties of proteins was developed. The method uses *z*-descriptors to represent amino acids in the protein sequences, and an ACC transformation for conversion of proteins into uniform vectors. The *k*NN clustering method showed the best performance among the other algorithms for classification tested in this study. The *k*NN algorithm was optimized and its performance was compared to the other web servers for allergenicity prediction. The algorithm was implemented on a web server, named AllerTOP, freely accessible *via *http://www.pharmfac.net/allertop. Apart from allergenicity, AllerTOP is able to predict the route of exposure of the allergen of interest.

## Methods

### Protein datasets

A set of 684 food, 1156 inhalant and 555 venom or salivary toxin allergens was collected from the CSL (Central Science Laboratory) allergen database (http://allergen.csl.gov.uk), the FARRP (Food Allergen Research and Resource Program) allergen database (http://www.allergenonline.org) and SDAP (Structural Database of Allergenic Proteins) (http://fermi.utmb.edu/SDAP/sdap_man.html). Twenty two of the food allergens were also toxins, 16 food allergens were also inhalant and 147 inhalant allergens were also toxins. A local database containing proteins of the allergen species was created from the NCBI database (http://fermi.utmb.edu/SDAP/sdap_man.html). It was used to construct a set of non-allergens that mirror the characteristics and origins of the allergen set. Non-allergens from the same species were selected after BLAST search towards each allergen. Non-allergens were selected as proteins with no sequence identity to known allergens, at an E-value of 0.001. In cases of insufficient numbers of non-allergens for a species in NCBI, a non-allergenic protein from the allergen genus or family was chosen. In cases of insufficient numbers of non-allergens from the allergen genus or family, human proteins were chosen randomly to fill the set. The total set of proteins used in the present study consisted of 2,210 allergens and 2,210 non-allergens.

### Presentation of protein sequences by *z*-descriptors and auto-cross covariance (ACC) transformation

In 1987, Hellberg and collaborators [23] derived the *z*-descriptors by principal component analysis on 29 principal physicochemical properties of amino acids. The hydrophobicity dominates in first principal component (*z_1_*), molecular size - in the second (*z_2_*), and polarity - in the third (*z_3_*). The *z*-values quantify the structural variations within a series of related proteins. In the present study the *z_1_*, *z_2 _*and *z_3 _*descriptors were used to describe the protein sequences.

Auto-cross covariance (ACC) transformation [18] was used in the present study in order to uniform the length of proteins. Two parameters - auto-covariance *Ajj(l) *and cross-covariance *Cjk(l) *- were calculated according to Eqs. (1) and (2), respectively:

(1) $$A_{jj}\left(l\right)=\overset{n-l}{\underset{i}{\sum }}\frac{Z_{j,i}\times Z_{j,i+1}}{n-l}$$

(2) $$C_{jk}\left(l\right)=\overset{n-l}{\underset{i}{\sum }}\frac{Z_{j,i}\times Z_{k,i+1}}{n-l}$$

Indices *j *and *k *refers to the *z*-descriptors (*j *= 1-3, *k *= 1-3, *j *≠ *k*), *n *is the number of amino acids in a sequence, index *i *ponts the amino acid position (*i *= 1, 2, ..., *n*) and *l *is the lag (*l *= 1, 2, ..., *L*). As only the influence of close amino acid proximity was investigated, short lags (*L *= 5) were chosen. The subsets of antigens and non-antigens were transformed into matrices with 45 variables (3^2 ^× 5) each.

### Machine learning methods for classification used in the study

The total set of allergens and non-allergens was subjected to two-class discriminant analysis by partial least squares (DA-PLS) using SIMCA-P 8.0 [26]. The optimum number of components was selected by adding components until the next added component explained less than 10% of the variance.

*K *nearest neighbours (*k*NN) and logistic regression (LR) algorithms were applied as implemented in python scripts based on the Biopython module [27]. The Naïve Bayes (NB) and decision tree (DT) algorithms were applied to the training set after the ACC transformation of sequences using WEKA Data Mining Software [28].

### Evaluation of performance

The correctly predicted allergens and non-allergens were defined as true positives (TP) and true negatives (TN), respectively. The incorrectly predicted allergen and non-allergens were defined as false negatives (FN) and false positives (FP), respectively. *Sensitivity *[TP/(TP + FN)], *specificity *[FP/(TN + FP)], *positive predictive value *(*ppv*) [TP/(TP + FP)] and *F1 *[2*sensitivity*ppv/(*sensitivity *+ *ppv*)] were calculated at threshold 0.5. The *area under ROC curve AUC *of the models also was calculated [29].

### Web servers for allergenicity prediction

AllerHunter (http://tiger.dbs.nus.edu.sg/AllerHunter) is a cross-reactive allergen prediction program built on a combination of Support Vector Machine (SVM) and pairwise sequence similarity [24]. Each proteins sequence in the training set is vectorized by performing sequence alignment and BLAST against all other members of the training set. The protein sequences are represented as vectors consisted of similarity scores for each pair of proteins in the training set.

AlgPred (http://imtech.res.in/raghava/algpred) predicts allergens by applying four different methods: MEME/MAST motif search (Algpred MEME), SVM-based classification of allergens and non-allergens by single amino acid composition (Algpred aa) and by dipeptide composition (Algpred dipep), and BLAST search against allergen representative peptides (Algpred ARP). MEME is a tool for discovering motifs in a group of related protein sequences. MAST searches in biological sequence databases for sequences that contain one or more groups of known motifs. Single amino acid composition gives the fraction of each amino acid in a protein. Dipeptide composition is used to encapsulate the global information about each protein sequence and gives a fixed pattern length of 400 (20 × 20). The BLAST search is performed against a set containing 24 amino acid long peptides, so called Allergen Representative Peptides (ARP), and finds proteins with high similarity to allergenic proteins [15].

## Competing interests

The authors declare that they have no competing interests.

## Authors' contributions

IrDo designed and supervised the study and drafted the manuscript. IvDi derived and validated the models, and designed the AllerTOP page. DRF advised on the study and helped with the writing of the manuscript. All authors revised and approved its final version.

## Supplementary Material

Additional file 1 **Additional file **1. Excel file. Click here for file

## Declarations

This article has been published as part of *BMC Bioinformatics *Volume 14 Supplement 6, 2013: Selected articles from the 10th International Conference on Artificial Immune Systems (ICARIS). The full contents of the supplement are available online at http://www.biomedcentral.com/bmcbioinformatics/supplements/14/S6.
